# Clinical efficacy of electroacupuncture antagonistic muscles combined with rehabilitation training in the treatment of spastic hemiplegia after stroke: a systematic review and meta-analysis of randomized controlled trials

**DOI:** 10.3389/fneur.2025.1634845

**Published:** 2025-08-08

**Authors:** Jingyue Liu, Xiang Meng, Yana He, Hailun Jiang, Jieying Zhang, Jinyuan Shi, Jingshu Zhang, Menglong Zhang, Fei Cai, Shizhe Deng, Jiangwei Shi, Zhihong Meng

**Affiliations:** ^1^National Clinical Research Center for Chinese Medicine Acupuncture and Moxibustion, Tianjin, China; ^2^First Teaching Hospital of Tianjin University of Traditional Chinese Medicine, Tianjin, China; ^3^Tianjin University of Traditional Chinese Medicine, Tianjin, China; ^4^Shanghai University of Traditional Chinese Medicine, Shanghai, China

**Keywords:** spastic hemiplegia, antagonistic muscles, rehabilitation training, systematic review, meta-analysis, electroacupuncture

## Abstract

**Objective:**

Antagonistic muscles rehabilitation training has been extensively utilized in the rehabilitation of patients with spastic hemiplegia. With the increasing acceptance and application of acupuncture, numerous scholars have discovered that incorporating electroacupuncture combined with antagonistic muscles rehabilitation training can enhance the treatment for spastic hemiplegia after stroke. The objective of this study is quantify the clinical efficacy of electroacupuncture in treating spastic hemiplegia after stroke on the basis of antagonistic muscles rehabilitation training, provide insights for future clinical treatments and scientific investigations related to spastic hemiplegia.

**Methods:**

We searched eight Chinese and English databases to identify clinical randomized controlled trials investigating the efficacy of electroacupuncture antagonistic muscles combined with rehabilitation training for the treatment of spastic hemiplegia after stroke. The search period extended from the inception of each database up to April 4, 2025. Two researchers independently reviewed the literatures, extracted relevant data, and assessed the risk of bias using the Cochrane Risk of Bias Tool.

**Results:**

A total of 8 literatures were selected from an initial pool of 1,680 for the purpose of meta-analysis. The findings indicated that the combination of electroacupuncture antagonistic muscles with rehabilitation training significantly alleviates muscle spasms in hemiplegic limbs [MD = –0.52,95%CI (−0.91, −0.13), *p* = 0.36, *I*^2^ = 0%, *Z* = 2.6, *p* < 0.05], enhances daily living capabilities [MD = 6.31,95%CI (3.91,8.71), *p* = 0.02, *I*^2^ = 59%, *Z* = 5.15, *p* < 0.05], improve motor function [MD = 9.9,95%CI (8.25,11.55), *p* = 0.2, *I*^2^ = 33%, Z = 11.75, *p* < 0.05]. Furthermore, we discovered that when the wave type and frequency of electroacupuncture are low frequency intermittent waves, electroacupuncture antagonistic muscles combined with rehabilitation training can effectively improve the lower limb motor function of hemiplegic patients. [MD = 10.52,95%CI (8.66,12.37), *p* = 0.89, *I*^2^ = 0%, *Z* = 11.11, *p* < 0.05], and electroacupuncture combined with Bobath technique is better than combined with conventional rehabilitation training.

**Conclusion:**

The integration of electroacupuncture treatment with antagonist muscle rehabilitation training can effectively alleviate muscle spasms, reduce muscle tension, and enhance lower limb motor function as well as daily living abilities.

## 1 Introduction

Stroke ranks as the second leading cause of death globally (1). Research indicates that approximately one in four individuals globally will experience a stroke (2), and the population affected by strokes is increasing each year (3). When hemorrhage or infarction occurs in the cerebral cortex, internal capsule or basal ganglia, the damage to the cortical-reticular projection neurons, along with heightened excitability of the pontine reticular spinal cord bundle and diminished inhibitory influence from the medullary reticular spinal cord bundle, results in an increased excitability of the spinal cord motor neurons (4, 5), the patients will exhibit static deformities as well as joint contractures associated with static deformity (6), According to statistical data, approximately 17.0 to 42.6% of stroke patients are likely to experience spastic hemiplegia (7), The spasticity of the limbs may be more pronounced in younger patients (8). Although spasmodic muscles can sustain the tone of the limbs to facilitating standing and walking, prolonged limb spasms may lead to permanent joint contractures and muscle atrophy. Therefore, if the spasmodic condition of the limb is not addressed in time, it will lead to increased pessimism among patients. This not only diminishes their adherence to rehabilitation efforts but also reduces the capacity for participation in daily activities and social engagement (9, 10), placing significant strains on healthcare systems and socio-economic structures (11).

The patients diagnosed with spastic hemiparesis primarily exhibit spasticity in the flexor muscles of the upper limbs, as well as spasticity in the extensor muscles of the lower limbs (12), spasticity is observed in the flexor muscle groups located on the anterior and medial aspects of the upper limbs. This includes muscles such as the pectoralis major, latissimus dorsi, vastus lateralis, brachialis, brachioradialis, and rotator cuff muscles responsible for elbow flexion. In addition, spasticity affects muscle groups in the lower extremities, primarily within the anterior-inferior thigh region and posterior calf area. This encompasses muscles such as the quadriceps, adductor group, gastrocnemius, and soleus (13). During treatment, it is essential to harmonize the tone of both active and antagonist muscle groups. This involves enhancing the lateral extensor muscle groups of the upper extremity while simultaneously strengthening the flexor muscle groups of the lower extremity. Such an approach promotes the emergence of limb separation movements and facilitates the establishment of normal movement patterns. Various rehabilitation modalities—including occupational therapy, plyometrics, and joint mobility training—can significantly improve the function of passive structures such as bone assemblies, joint surfaces, and ligaments, as well as active structures like muscle-tendon complexes. These interventions address issues related to mechanical alignment or kinematic alignment anomalies, thereby increasing antagonist muscle strength and inhibiting pathological reflexes. While straightforward rehabilitation training can effectively alleviate limb spasticity, it necessitates significant investment in healthcare personnel, funding, and equipment. Furthermore, in low-income countries and regions, patients may be reluctant to engage with treatment due to the prolonged duration of therapy, slow progress in results, and high associated costs.

With the growing interdisciplinary connections and the recent integration of therapeutic approaches, the efficacy of acupuncture in conjunction with modern rehabilitation techniques for treating post-stroke hemiplegia has increasingly emerged as a prominent area of research (14). Due to its simplicity and efficacy (15), acupuncture is endorsed by the World Health Organization as a complementary and alternative treatment for stroke and its sequelae (16). The application of electroacupuncture, a therapeutic approach that incorporates pulsed electrical stimulation alongside the deqi from millimeter acupuncture (17), offers several advantages. These include precise parameter settings, quantifiable intensity and frequency of stimulation, and consistent levels of acupuncture intervention. Such characteristics can significantly aid in the formulation of a clinically standardized treatment strategy for spastic hemiparesis following stroke. It has been demonstrated that electroacupuncture induces excitatory contractions by stimulating the active muscle, leading to impulse signals being transmitted to the spinal cord via type Ia afferent nerves. This process facilitates the inhibition of corresponding antagonist muscles and alleviates muscle spasms (18). In accordance with the principles of human biomechanics, the movement patterns and neuroanatomical considerations associated with spastic limbs, electroacupuncture antagonist muscles combined with rehabilitation training can significantly enhance limb function and improve daily living capabilities. Furthermore, research has demonstrated that acupuncture can treat spastic hemiparesis through various mechanisms, including improving blood perfusion, reducing neuroinflammation, regulating neuroplasticity (19), remodeling corticospinal tracts, repairing descending motor pathways (20, 21), inhibiting the release and transport of excitatory neurotransmitters to prevent neuronal excitotoxicity (22, 23), decreasing the excitability of spinal cord anterior horn motoneurons, and modifying muscle structure to improve muscular motility in multiple ways (24). The purpose of this paper is to conduct a meta-analysis of the improvement of electroacupuncture antagonistic muscles combined with rehabilitation training on patients with hemiplegia after stroke.to provide the foundation for future in-depth investigations into the effects of electroacupuncture antagonist muscles alongside rehabilitation training therapy on limb function recovery post-stroke. This study has been registered in PROSPERO (No: CRD420251045251).

## 2 Methods

### 2.1 Study selection

We searched across eight Chinese and English databases, including PubMed, Cochrane Library, Embase, Web of Science, China Biology Medicine disc (CBM), China National Knowledge Infrastructure (CNKI), China Science Periodical Database (CSPD), and the Chinese Citation Database (CCD). The search period for each database extended from its inception until April 4, 2025. The languages utilized in the search were restricted to Chinese and English. The search strategy employed a combination of subject terms and free-text keywords. The Chinese search terms included “spastic hemiplegia,” “hemiplegia,” “hemiplegia with stroke,” “hemiplegia with hemiparesis,” “post-stroke hemiplegia,” “dystonia enhancement,” “electro-acupuncture,” “electro-acupuncture interventions,” “acupuncture plus electro-acupuncture,” “electro-acupuncture stimulation,” “conventional electro-acupuncture,” “electro-acupuncture treatment,” as well as various forms of randomized controlled trials such as RCTs and related terminology. The English search terms comprised “hemiplegia,” “hemiplegias,” “electroacupuncture,” “randomized controlled trial,” “randomized,” and “placebo.” To develop effective search strategies tailored to the specific requirements and characteristics of different databases in both languages, we applied the PICOS principle while combining spastic hemiplegia subject terms with relevant free-text keywords. This approach aimed to yield comprehensive primary literature on the topic. All search strategies were finalized following multiple iterations of searches. We imported the retrieved titles of Chinese and English literature into NoteExpress software to assess their weights and eliminate duplicates. Two researchers independently reviewed the titles and abstracts, manually re-evaluated the weights, and screened the literatures according to predefined inclusion criteria. Subsequently, both researchers independently downloaded the literatures that met these criteria, read the full texts, and conducted a further screening based on the same inclusion standards. Ultimately, we determined which pieces of literature would be included in our study. Finally, both researchers cross-verified their screening processes; in cases of discrepancies, they engaged in discussions until reached agreement.

### 2.2 Inclusion criteria

#### 2.2.1 Type of article

Randomized controlled clinical trials of electroacupuncture antagonistic muscles combined with rehabilitation training in the treatment of spastic hemiplegia after stroke.

#### 2.2.2 Research participants

Diagnosed with cerebral infarction or cerebral hemorrhage in Western medicine; accompanied by limb motor dysfunction; there are no restrictions regarding age, gender, or race.

#### 2.2.3 Intervention

The observation group received electroacupuncture antagonist muscles, in conjunction with rehabilitation training and basic treatment. In contrast, the control group underwent rehabilitation training along with basic treatment. Both groups followed an identical course of treatment. The conventional basic treatment employed in Western medicine encompassed antiplatelet aggregation therapy, antihypertensive measures, lipid-lowering interventions, and enhancements to cerebral metabolism, among others. The dosage and duration of these treatments were not subject to restrictions.

#### 2.2.4 Quality assessment criteria

The methodological quality of the included studies was assessed using the Cochrane Collaboration’s risk of bias tool (Risk of Bias Tool). This evaluation encompassed seven key items: randomized sequence generation (selection bias), allocation concealment (selection bias), blinding of subjects and investigators (implementation bias), blinding of outcome evaluators (measurement bias), incomplete outcome data (loss to follow-up bias), selective reporting of outcomes (reporting bias), and other biases. Each item was classified as “low risk,” “unclear,” or “high risk.” To ensure the integrity and quality of the literatures, two independent investigators evaluated the risk of bias for each study. They subsequently discussed their findings and reached a consensus regarding the overall assessment of risk.

#### 2.2.5 Criteria for exclusion

(1) Duplicate literatures; (2) Systematic reviews, commentaries, letters, meeting abstracts, and research protocols; (3) Literatures derived from non-randomized controlled trials; (4) Interventions that do not align with the study criteria; (5) Absence of relevant outcome indicators or inability to extract valid data; (6) Literatures for which the full text is inaccessible; (7) Non clinical trials.

#### 2.2.6 Data extraction

Two researchers independently entered the information from the final included literatures into Microsoft Excel, following the data extraction form. They subsequently cross-checked the entered data with each other and engaged in discussions to resolve any discrepancies. The extracted data encompassed: (1) the name of the researcher, title of the literature, year of publication, sample size, shedding rates, adverse reactions, and other relevant details; (2) patient demographics including age, gender, duration of disease, duration of treatment, interventions administered, controls utilized; and (3) assessments regarding the risk of bias in randomized controlled trials. (4) The outcome measures included the primary endpoint: overall clinical effectiveness, and secondary endpoints such as the simplified Fugl-Meyer motor function rating scale. Additionally, the assessment tools utilized were the Fugl-Meyer Assessment (FMA), Barthel Index (BI), and Modified Ashworth Scale (MAS). In cases where study data were inadequately described or information was lacking in the literature, we were contacted via email for clarification; otherwise, those literatures were excluded from consideration.

#### 2.2.7 Statistical analysis

The software package provided by the Cochrane Collaboration (Review Manager 5.3) was utilized for conducting heterogeneity tests, evaluating publication bias, performing subgroup analyses, and executing sensitivity analyses. Measurement data were analyzed using mean difference (MD), expressed with a 95% confidence interval (CI), along with the I^2^ test to assess heterogeneity. When the statistical results indicated low heterogeneity (0% ≤ *I*^2^ < 49%), a fixed-effects model was employed; conversely, when inter-study heterogeneity was substantial (*I*^2^ ≥ 50%), a random-effects model was applied to aggregate effect sizes. Additionally, we examined the sources of heterogeneity and conducted subgroup analyses based on potential factors contributing to this variability (rehabilitation measures, duration of treatment, disease duration). Sensitivity analysis was performed as necessary to evaluate the robustness of combined effect size estimates in light of study outcomes. In cases where excessive heterogeneity precluded identification of its source, descriptive analysis was adopted instead. Funnel plots were drawn for the main outcome indicators to assess publication bias.

## 3 Results

### 3.1 Literature search

**Figure 1:**
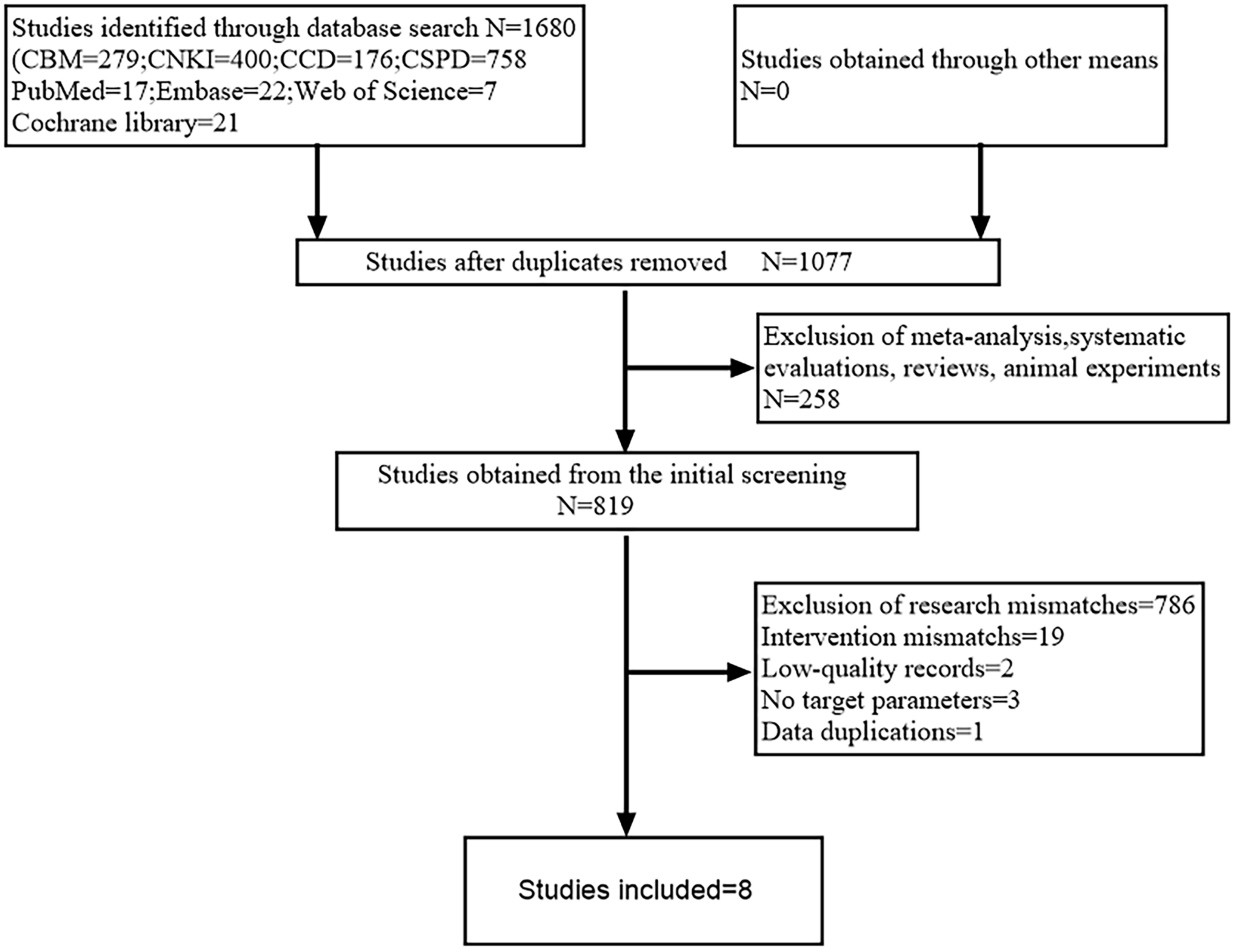
Literature screening flowchart.

The results yielded a total of 1,680 relevant pieces of literature. Specifically, there were 17 literatures identified in PubMed, 21 literatures in the Cochrane Library, 22 literatures in Embase, 7 literatures in Web of Science, 279 literatures in CBM, 400 literatures in CNKI, 758 literatures in CSPD, and 176 literatures in CCD. Following a thorough review using NoteExpress software and manual verification for duplicates, we excluded a total of 603 duplicate entries. Additionally, we eliminated 258 literatures based on an assessment of their titles and abstracts. After reviewing the full texts, we further excluded: 786 literatures due to mismatches with study content, 19 literatures because of discrepancies regarding interventions, 2 literatures from low-quality journals, 3 literatures that lacked target parameters, and finally, 1 literature was removed due to data duplication. Ultimately, eight studies were included in the final selection process (25–32). The screening process is illustrated in Figure 1.

### 3.2 Fundamental characteristics of the included literature

We included a total of 8 randomized controlled trials, comprising 2 dissertations and 6 published papers. There are 584 cases, with 293 participants in the intervention group and 291 participants in the control group. The smallest sample size among the studies was 40 cases, while the largest was 80 cases. Six studies reported on the waveforms and frequencies associated with electroacupuncture; all eight studies documented treatment duration, which ranged from a minimum of 3 weeks to a maximum of 16 weeks. The included studies featured two types of rehabilitation therapies: five focused on conventional rehabilitation training and three utilized the Bobath technique. The fundamental characteristics of these studies are presented in Table 1.

**Table 1:** Fundamental characteristics of the included literature.

References	Sample size experimental/control	Method of intervention	Electroacupuncture stimulation intensity	Comparison	Outcome	Frequency	Retained	Course
Geng (28)	20/20	Electroacupuncture + Routine rehabilitation	Tolerated by the patients	Routine rehabilitation	1,2,4	Once/d 7 times/week	30 min	3 weeks
Hu (29)	30/30	Electroacupuncture + Bobath	Sparing waveforms	Bobath	1,2,3	Once/d 6 times/week	40 min	8 weeks
Ren (25)	34/34	Electroacupuncture + Routine rehabilitation	High frequency 60times/s	Routine rehabilitation	1,3	Once/d 7 times/week	30 min	30d
Wang (2006)	24/22	Electroacupuncture + Bobath	The needle and the muscle appear to be slight trembling	Bobath	1,2	Once/d 6 times/week	30 min	16 weeks
Ye (31)	40/40	Electroacupuncture + Routine rehabilitation	Low frequency intermittent wave	Routine rehabilitation	1,2,3	Once/d 7 times/week	30 min	30d
Zhang (27)	38/38	Electroacupuncture + Routine rehabilitation	Low frequency intermittent wave	Routine Rehabilitation	1,2,3	Once/d 7 times/week	30 min	30d
Liu (30)	40/40	Electroacupuncture + Bobath	Low frequency intermittent wave	Bobath	1,3,4	Once/d 7 times/week	30 min	30d
Xu (32)	30/30	Electroacupuncture + Routine rehabilitation	High frequency 100 Hz	Routine rehabilitation	1	Once/d 7 times/week	30 min	24 weeks

1, Overall clinical effectiveness 2, Barthel Index 3, Fugl-Meyer Assessment 4, Modified Ashworth Scale.

### 3.3 Evaluation results of the methodological quality of the included studies

**Figure 2:**
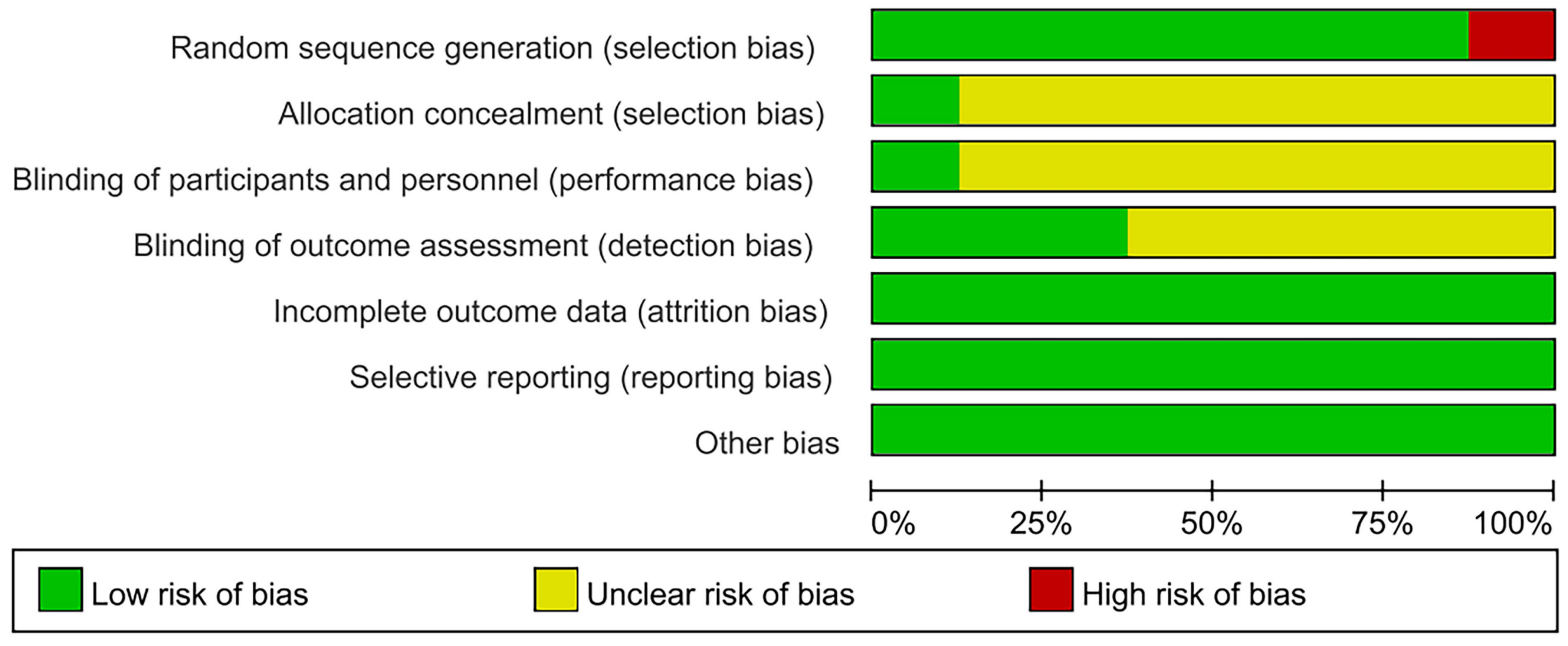
Bias of risk graph.

**Figure 3:**
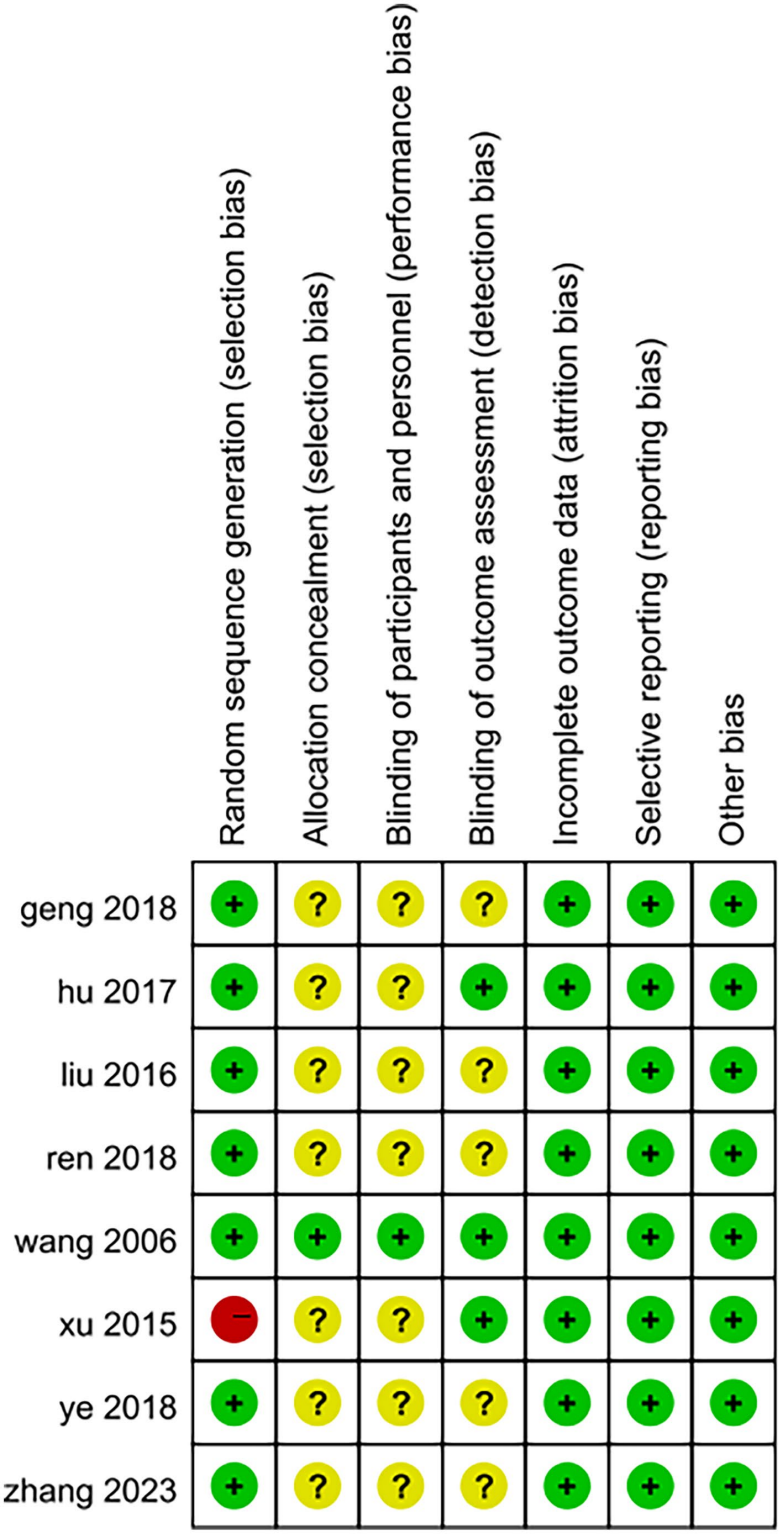
Bias of risk summary.

The quality of the included studies was evaluated according to Cochrane criteria: (1) Random sequence generation: three studies utilized randomized numeric tables (29–31), two employed randomized sampling (25, 27), and one study implemented randomized serial numbers (26); One study (28) merely mentioned randomization without specifying the method of allocation; however, the baseline characteristics in terms of demographic information and clinical features among the included subjects were comparable, leading to an assessment of “low risk.” Conversely, one study (32) grouped patients based on their order of hospital attendance, which constituted a semi-randomized approach and was assessed as “high risk.” (2) Allocation concealment: one study (26) reported on allocation concealment and was therefore rated as “low risk,” while seven studies did not provide this information, resulting in an assessment of “unclear.” (3) Blinding of outcome assessors: three studies (26, 29, 32) blinded outcome assessors and were classified as “low risk”; five studies did not specify whether blinding occurred for outcome assessors and were deemed “unclear.” (4) Blinding of the included population and experimental personnel: One study (26) implemented blinding for both the included population and experimental personnel, resulting in an assessment of “low risk.” In contrast, seven studies did not specify whether blinding was applied to the included population and experimental personnel; therefore, these were assessed as having an “unclear” risk. (5) Completeness of outcome data: One study (26) reported instances of dislodgement and loss to follow-up. Specifically, one patient experienced dislodgement and subsequently lost to follow-up (the exact reason remains unknown), while another patient was excluded from the results due to the use of muscarinic agents during the treatment period. Conversely, seven studies reported no occurrences of dislodgement or loss to follow-up; all were deemed complete and assessed as “low risk.” (6) Selective Reporting: The results section of all studies in the literature aligns with the methodology section. Given that there is no evidence of selective reporting, this aspect has been assessed as presenting a “low risk.” (7) Other Sources of Bias: All studies was standardized, and demographic data along with clinical characteristics of all patients were comparable at baseline. Furthermore, no other sources of bias that could potentially affect the results were identified; therefore, this risk has also been evaluated as “low.” The outcomes of the risk of bias assessment for the included studies are presented in Figures 2, 3.

### 3.4 Results of the meta-analysis

#### 3.4.1 Overall clinical effectiveness

**Figure 4:**
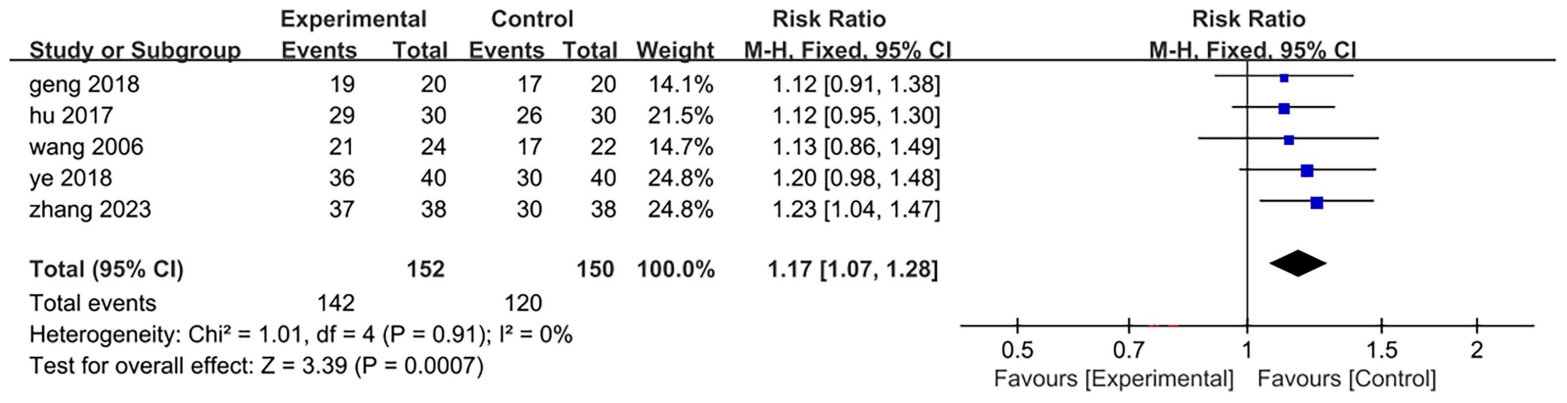
Forest plot of the changes of the overall clinical efficacy.

Five of the eight included studies (26–29, 31) reported overall clinical efficacy rates, encompassing a total sample size of 302 cases: 152 in the treatment group and 150 in the control group. The results of the heterogeneity test indicated *p* = 0.91 and *I*^2^ = 0%, suggesting that the heterogeneity among the literatures included in this analysis was not statistically significant. Consequently, a fixed-effect model was employed to combine effect sizes. The meta-analysis results revealed a relative risk (RR) of 1.17 with a 95% confidence interval (CI) ranging from 1.07 to 1.28, demonstrating statistical significance (*Z* = 3.39, *p* = 0.0007 < 0.001). This indicates that the overall clinical efficacy for patients in the treatment group surpassed that of those in the control group; specifically, electroacupuncture intervention targeting antagonist muscles combined with rehabilitation training proved more effective than rehabilitation training alone[RR = 1.17,95%CI(1.07,1.28), *Z* = 3.39, *p* = 0.0007 < 0.001]. Detailed results are illustrated in Figure 4.

#### 3.4.2 Assessments for bias

**Figure 5:**
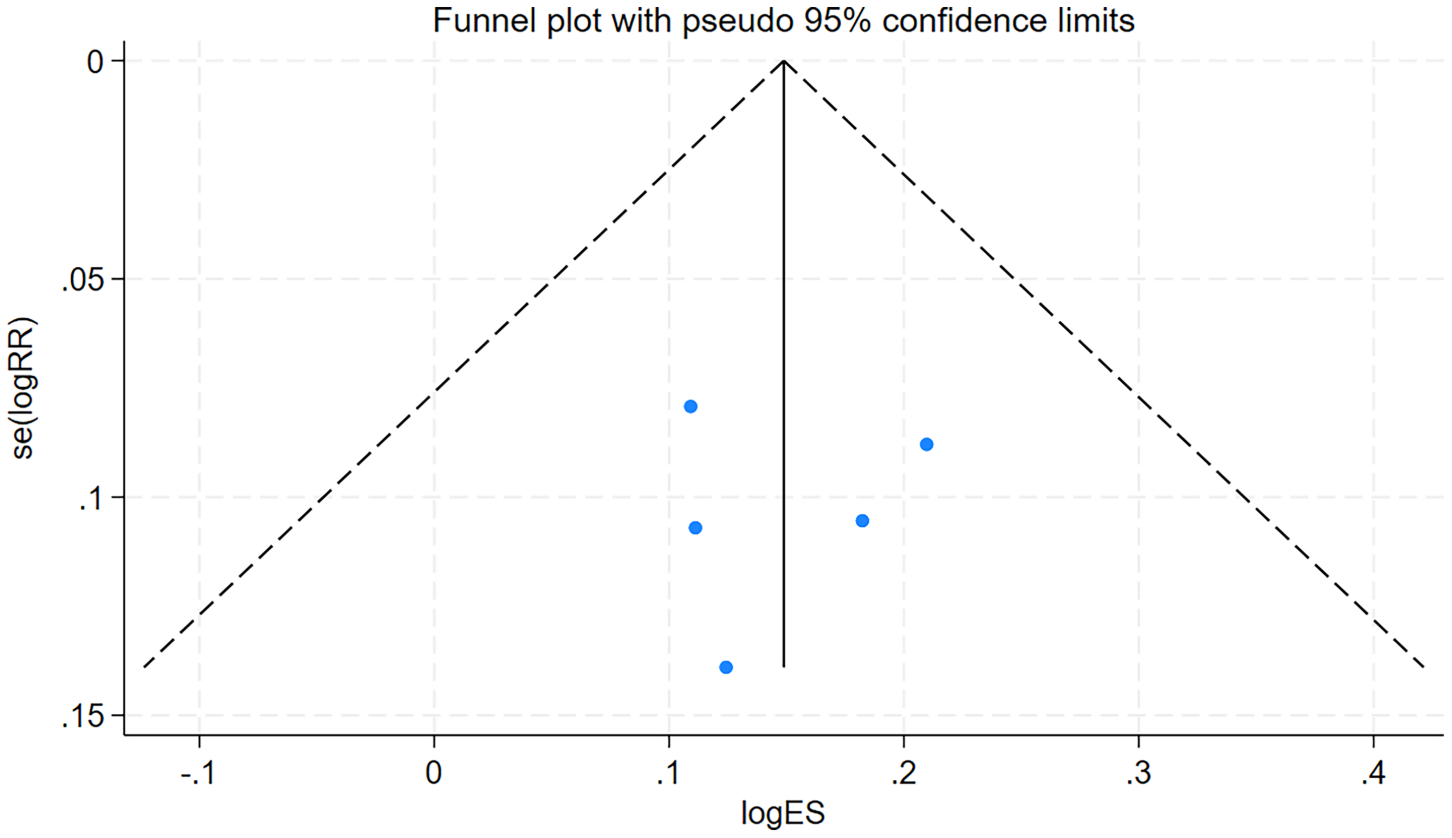
Funnel plot of the overall clinical efficacy.

The total clinical efficacy rate of the included studies serves as the primary index. The funnel plot derived from the five studies included in this analysis was evaluated using Stata 18, the outcome of the test is *p* = 0.936 (*p* > 0.05). This indicates that the funnel plot is symmetrical and free from publication bias. The detailed results are presented in Figure 5.

#### 3.4.3 Barthel index

**Figure 6:**
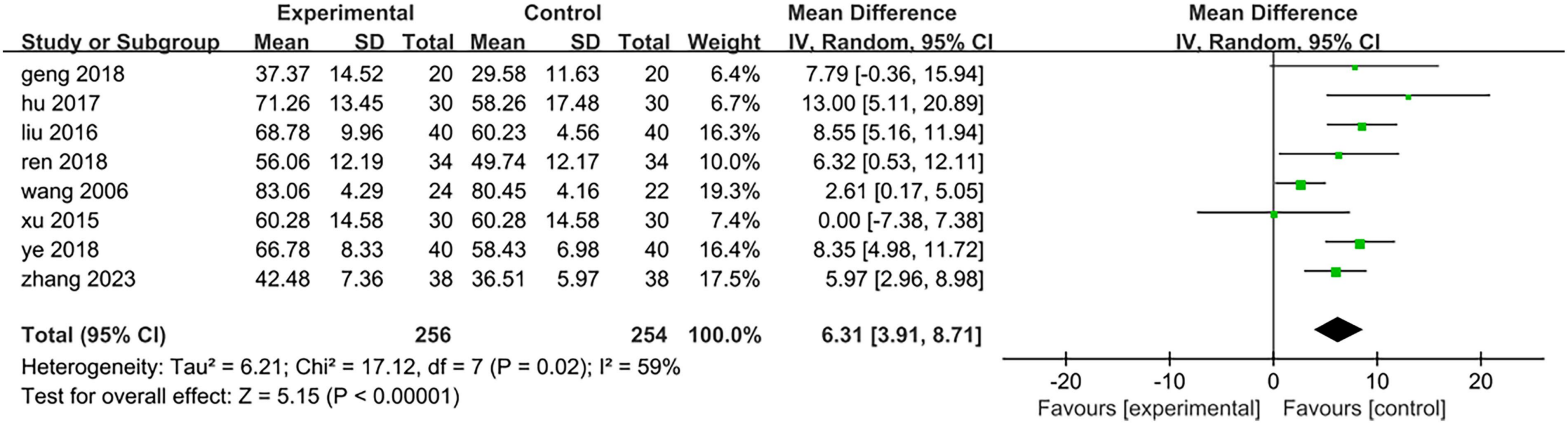
Forest plot of the changes of the Barthel Index.

**Figure 7:**
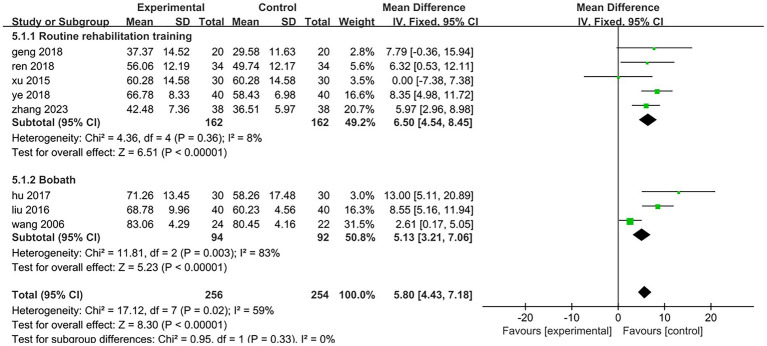
Forest plot of the subgroup analysis of rehabilitation interventions.

**Figure 8:**
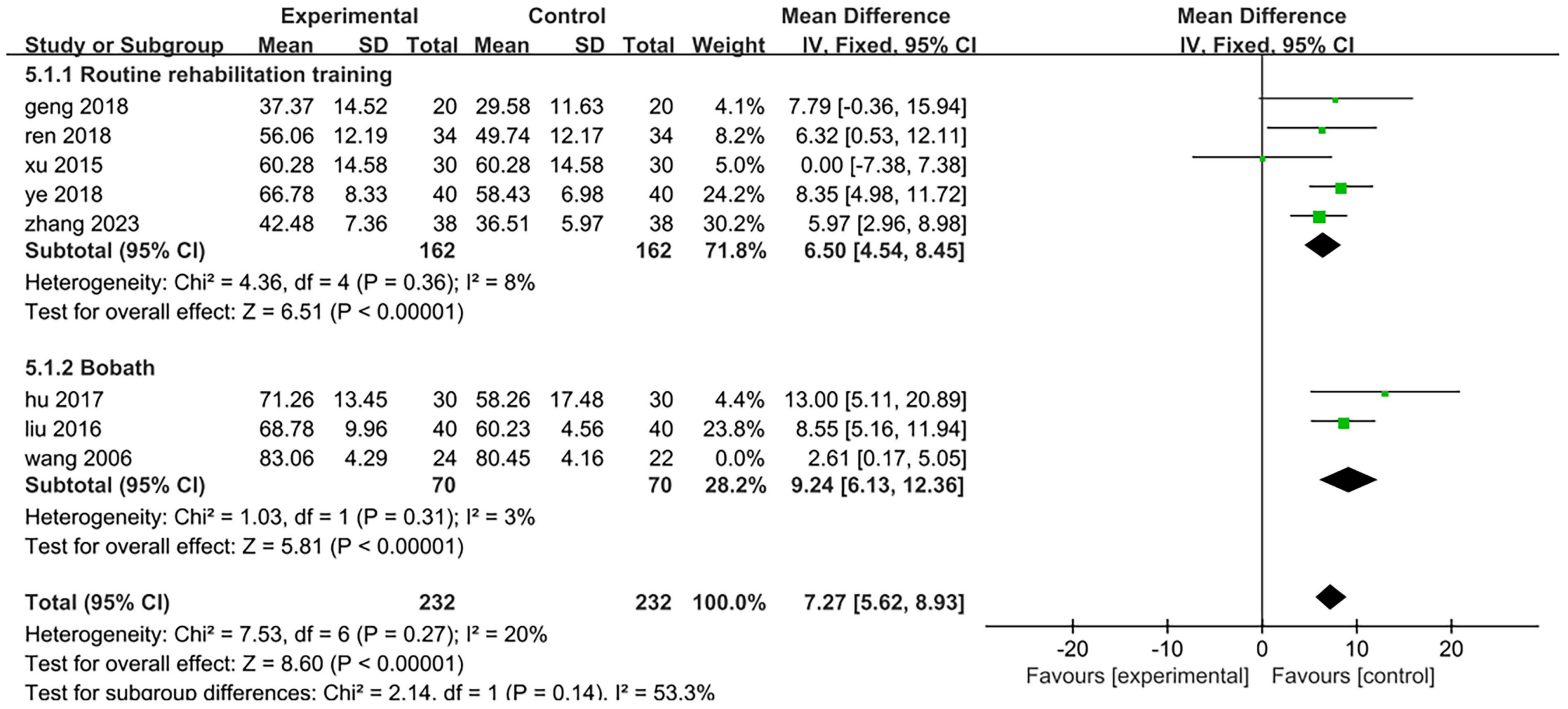
Forest plot of the subgroup analysis of electroacupuncture antagonistic muscles combined with Bobath technique.

All eight included studies assessed the improvement in daily mobility between the two groups before and after treatment. Four studies utilized the Barthel Index, while the other four employed the Modified Barthel Index. The total number of participants was 510, with 256 in the treatment group and 254 in the control group. The heterogeneity test yielded a result of *p* = 0.02 and *I*^2^ = 59%, indicating a high degree of heterogeneity; thus, a random effects model was applied for analysis. The meta-analysis revealed a mean difference (MD) of 6.31 with a 95% confidence interval (CI) ranging from 3.91 to 8.71, which was statistically significant (*Z* = 5.15, *p* < 0.05). This suggests that electroacupuncture targeting antagonist muscles combined with rehabilitation training can effectively enhance patients’ daily living abilities [MD = 6.31,95%CI (3.91,8.71), *Z* = 5.15, *p* < 0.05], as illustrated in Figure 6. To analyze the rehabilitation interventions within subgroups, five studies (25, 27, 28, 31, 32) involving a total of 324 cases employed conventional rehabilitation training. The results of the heterogeneity test indicated *p* = 0.36 and *I*^2^ = 8%, suggesting low heterogeneity. Consequently, data were combined using a fixed-effect model. The meta-analysis revealed a mean difference (MD) of 6.5 with a 95% confidence interval (CI) ranging from 4.54 to 8.45, which was statistically significant (*Z* = 6.51, *p* < 0.01). These findings suggest that electroacupuncture antagonist muscles in conjunction with routine rehabilitation training significantly enhances the daily living abilities of hemiplegic patients [MD = 6.5,95%CI (4.54,8.45), *Z* = 6.51, *p* < 0.01]; specific results are illustrated in Figure 7. The rehabilitation interventions in three studies (26, 29, 30) employed the Bobath technique. The heterogeneity test yielded a result of *p* < 0.01 and *I*^2^ = 83%, indicating substantial heterogeneity; specific results are illustrated in Figure 7. In one study (26) (Wang, 2006, *n* = 46), the data recording period was extended to 16 weeks, which was longer than the treatment duration in the other two trials. Upon excluding this study from the heterogeneity analysis, we found that *p* = 0.31 and *I*^2^ = 3%, suggesting low levels of heterogeneity. For the remaining two studies (29, 30) (*n* = 140), data were combined using a fixed-effects model for meta-analysis. The results indicated a mean difference (MD) of 9.24 with a confidence interval (CI) of 95% (6.13,12.36), achieving statistical significance (*Z* = 5.81, *p* < 0.01). This suggests that within an eight-week period, electroacupuncture antagonist muscles in conjunction with the Bobath technique is effective in enhancing daily living abilities among hemiplegic patients [MD = 9.24,95%CI (6.13,12.36), *Z* = 5.81, *p* < 0.01]; detailed findings are presented in Figure 8.

#### 3.4.4 Assessments for bias

**Figure 9:**
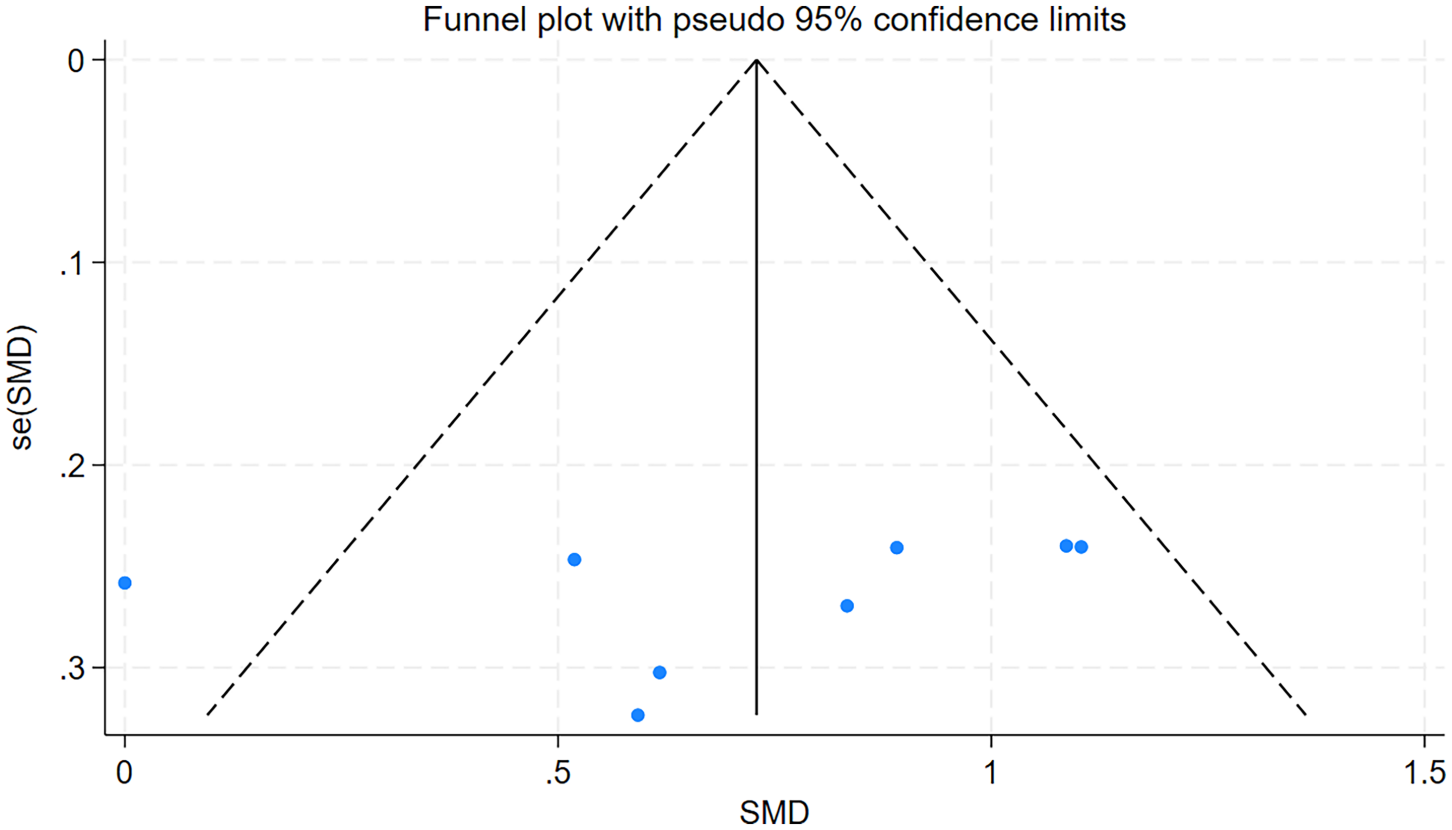
Funnel plot of the Barthel Index.

The funnel plot was drawn by Stata18 to test the 8 articles included in this study, *p* = 0.421 > 0.05, suggesting that there was no publication bias. The detailed results are presented in Figure 9.

#### 3.4.5 Fugl-Meyer assessment

**Figure 10:**
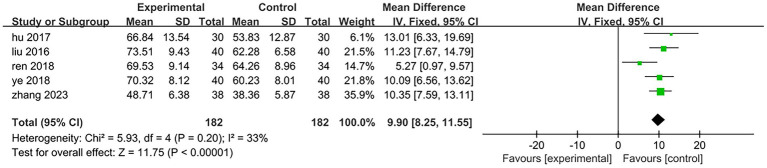
Forest plot of the changes of the Fugl-Meyer Assessment.

**Figure 11:**
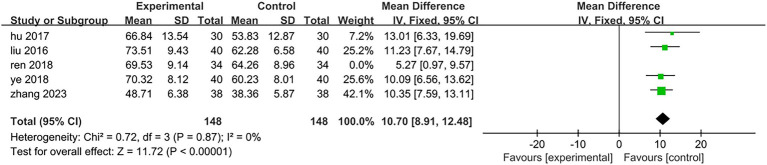
Forest plot of the changes of the Fugl-Meyer Assessment after heterogeneity analysis.

**Figure 12:**
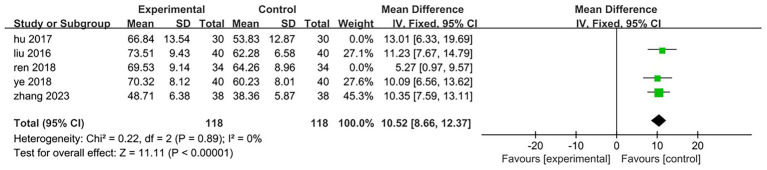
Forest plot of the low-frequency intermittent wave electroacupuncture antagonistic muscle combined with rehabilitation training to improve lower limb motor function.

Five of the eight included studies assessed motor function. Three studies (25, 27, 31) utilized the Fugl-Meyer Motor Function Rating Scale, while two studies (29, 30) employed the Simplified Fugl-Meyer Motor Function Rating Scale. The total sample size comprised 182 participants, with 91 in the treatment group and 91 in the control group. The results of the heterogeneity test indicated *p* = 0.2 and *I*^2^ = 33%, suggesting that there was minimal statistical heterogeneity among the included literatures; thus, a fixed effect model was applied for analysis. The meta-analysis revealed a mean difference (MD) of 9.9 with a 95% confidence interval (CI) ranging from (8.25 to 11.55), which was statistically significant (*Z* = 11.75, *p* < 0.05). This indicates that patients in the treatment group exhibited superior lower limb motor function compared to those in the control group. It can be concluded that electroacupuncture targeting antagonist muscles combined with rehabilitation training significantly enhanced patients’ lower limb motor function relative to controls [MD = 9.9,95%CI (8.25,11.55), Z = 11.75, *p* < 0.05]; specific results are illustrated in Figure 10. One of the studies (25) (Ren 2018, 68 cases) included patients with a longer disease duration compared to those in other studies. Additionally, the rehabilitation training implemented in this trial is a personalized rehabilitation program, resulting in low consistency regarding the content of the rehabilitation training among the included patients. After excluding this study, the results of the heterogeneity test indicated *p* = 0.87 and *I*^2^ = 0%, suggesting that the heterogeneity among the remaining four studies included in this analysis was statistically insignificant. The specific results are illustrated in Figure 11. In the three studies analyzed, the electroacupuncture employed consisted of low-frequency intermittent waves. The parameters for electroacupuncture interventions—including the number of sessions, retention time, and treatment duration—were consistent across all studies. The heterogeneity test yielded *p* = 0.89 and *I*^2^ = 0%, indicating that there was minimal statistical heterogeneity among the three papers included in this analysis. The results of the meta-analysis demonstrated a mean difference (MD) of 10.52 with a 95% confidence interval (CI) ranging from 8.66 to 12.37, which is statistically significant (*Z* = 11.11, *p* < 0.05). This suggests that low-frequency intermittent wave electroacupuncture combined with rehabilitation training can effectively enhance lower limb motor function in hemiplegic patients on a daily basis [MD = 10.52,95%CI (8.66,12.37), *Z* = 11.11, *p* < 0.05]. Specific results are illustrated in Figure 12.

#### 3.4.6 Assessments for bias

**Figure 13:**
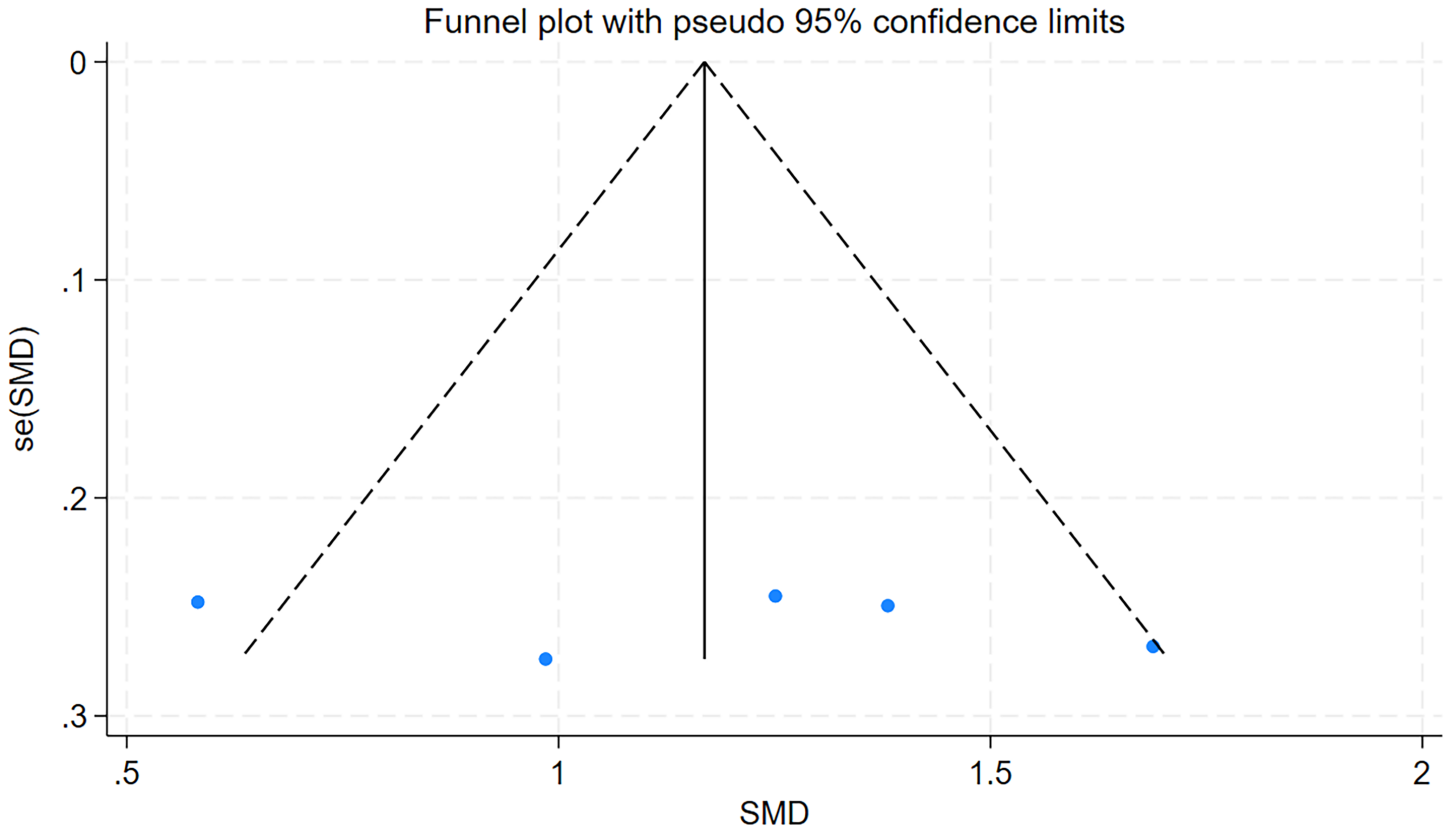
Funnel plot of the Fugl-Meyer assessment.

The funnel plot was drawn by Stata18 to test the 5 articles included in this study, *p* = 0.674 > 0.05, suggesting that there was no publication bias. The detailed results are presented in Figure 13.

#### 3.4.7 Modified Ashworth scale

**Figure 14:**
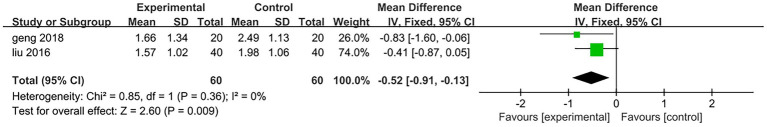
Forest plot of the changes of the Modified Ashworth Scale.

There are two studies (28, 30) among the eight included in this review that assessed the improvement in limb spasticity before and after treatment in both groups. The total sample size comprised 120 cases, with 60 participants in the treatment group and 60 in the control group. The results of the heterogeneity test indicated *p* = 0.36 and *I*^2^ = 0%, suggesting that there was no statistically significant heterogeneity among the included studies; thus, a fixed-effect model was employed for analysis. The meta-analysis revealed a mean difference (MD) of −0.52 with a 95% confidence interval (CI) ranging from −0.91 to −0.13, which was statistically significant (Z = 2.6, *p* = 0.009 < 0.05). This finding indicates that electroacupuncture combined with antagonist muscle rehabilitation training can effectively improve limb spasticity in patients with hemiplegia [MD = –0.52,95%CI(−0.91,–0.13), Z = 2.6, *p* < 0.05]. The specific results are illustrated in Figure 14.

#### 3.4.8 Security analysis

None of the eight studies reported any adverse effects, and the incidence of such effects is found to be low. One study (26) did mention instances of dislodgement and missed appointments; specifically, one patient experienced dislodgement and subsequently lost to follow-up (the exact cause remains unknown), while another patient was removed from the study due to the muscle relaxant during treatment.

## 4 Discussion

There remain areas for improvement in this study: (1) We have observed that low-frequency intermittent waveforms can effectively alleviate spasticity; however, the sample size of our study is limited. Future research should involve multi-center and large-sample studies to explore the differences in efficacy among various waveforms and frequencies in the treatment of hemiplegia. This will help confirm the optimal clinical conditions for different waveform frequencies in relation to Brunnstrom stage, as well as clarify protocols for spasticity staging corresponding to specific waveforms, frequencies, and intensities of electroacupuncture. (2) The literatures included in this study discusses electroacupuncture treatment following the sensation of deqi; however, only one study (29) provided the description of the performance of deqi. Furthermore, none of the studies elaborated on the specific techniques employed to achieve acupuncture deqi, as the experience of deqi primarily relies on patients’ subjective perceptions. There is considerable variability in how different patients perceive deqi (33), In future research, it is essential to standardize various factors related to needling operations, including frequency, duration, direction, and depth. Additionally, integrating multimodal perception fusion technologies and incorporating visual parameters such as needle movement amplitude alongside tactile characteristics like finger pressure (34) will help mitigate the impact of subjective variables associated with manipulation on test outcomes. (3) The primary outcome indicators included in this study exhibit a high degree of subjectivity (35), which results in a lack of standardization in the evaluation of clinical efficacy. Additionally, there is a scarcity of original data available for homogeneity analysis. In future research, objective indicators such as the touchdown area and maximum pressure peak across various regions of the plantar surface can be measured to assess the coordinated function of foot internal and external rotation, as well as lower limb skeletal joints. This approach will enable a scientific and objective evaluation of patients’ stride frequency, stride length, and support time. (4) The patients included in this study were all of the Asians, which is characterized by certain geographical limitations. With the gradual global acceptance of acupuncture and moxibustion (36), it is essential to conduct relevant randomized controlled trials involving different races in the future. Furthermore, the therapeutic effects of electroacupuncture antagonistic muscles combined with rehabilitation training should be extended to a broader patient population worldwide. Additionally, establishing a database for regional spastic paralysis across various regions will provide valuable data support for clinical treatment and scientific research. (5) The findings of this study indicate that electroacupuncture interventions antagonistic muscles can significantly alleviate spasm symptoms in patients. However, electroacupuncture can be applied to antagonistic muscles through various approaches, including the selection of starting and ending points of these muscles, tendon nodes, muscle movement points, and corresponding acupoints based on the specific antagonistic muscles involved (37). Nevertheless, the differences in efficacy and treatment mechanisms among these diverse intervention methods remain unclear. (6) This meta-analysis demonstrates that electroacupuncture combined with Bobath technique results in better outcomes than electroacupuncture combined with the conventional rehabilitation training. However, it is important to note that the sample size of patients included in this study is limited. Future research should focus on multi-center large-sample randomized controlled trials to further investigate the differences in efficacy of electroacupuncture combined with different rehabilitation training approaches to provide high-quality evidence. (7) Due to variations in the random allocation methods employed in the included studies, confounding factors such as demographic and disease characteristics may emerge. Furthermore, considering the difficulty of implementing a double-blind design in clinical studies of acupuncture, making it difficult to maintain blinding for participants, experimenters, and outcome assessors. This situation can lead to potential assessment bias from outcome evaluators. Additionally, it is difficult to quantify and exclude the influence of acupuncture placebo effects on participants and researchers. The limited sample sizes further restrict the statistical power of study findings, thereby increasing the likelihood that results may be affected by chance or outliers. All included studies were conducted at single centers involving Asian participants; thus, they do not adequately represent patients with spastic hemiplegia from other regions, which limits the generalizability of these findings. Moreover, the short duration of interventions is insufficient for effectively evaluating both long-term efficacy and safety of electroacupuncture combined with rehabilitation training for spastic hemiplegia post-stroke—this includes potential delayed adverse reactions. Future research should be designed as rigorous multicenter randomized controlled trials with large sample sizes. Stratified randomization methods should be utilized to group participants based on age, gender, and disease severity prior to their random assignment into intervention and control groups; this will help ensure balanced baseline characteristics such as age and disease severity. It is essential to select objective outcome measures that are resistant to interference while employing ANCOVA for correcting baseline values during statistical analyses. Additionally, extending both treatment duration and follow-up periods appropriately is necessary to elucidate the long-term efficacy and safety of electroacupuncture combined with rehabilitation training in treating spastic hemiplegia after stroke.

## 5 Suggestions for future development direction

(1) Due to physical dysfunction, social role change and other reasons, hemiplegic patients will face the pressure of economic burden (38), family burden and self-cognitive burden during the rehabilitation period, resulting in decreased happiness and increased loneliness (39). Effective emotional support and psychological counseling (such as Swanson care theory) can effectively alleviate depression (40), foster positive life experiences, enhance quality of life, and facilitate disease recovery. Research indicates that acupuncture may inhibit neuronal excitotoxicity by reducing glutamate (Glu) expression in the cerebral cortex while promoting gamma-aminobutyric acid (GABA) expression. This regulation of the ‘GABA-Glu’ balance can improve muscle spasms. The disruption of Glu/GABA homeostasis across various brain regions is recognized as a contributing factor to severe depressive disorders (41) and plays a role in the mechanisms underlying antidepressant efficacy (42). Therefore, in the future, it should carry out high-quality randomized controlled trials to explore the effect of electroacupuncture antagonistic muscle on patients with spastic hemiplegia and depression by improving ‘GABA-Glu ‘imbalance. (2) At present, the mechanism of electroacupuncture antagonistic muscles in the treatment of spastic hemiplegia predominantly is a single-mechanism approach. It is essential to investigate the effects of various parameters associated with antagonistic muscles electroacupuncture on different biological indices within the human body. This exploration should integrate techniques such as acupuncture manipulation quantification (43), modern systems biology, and mechanical biology. For instance, studies could focus on synaptic structure reconstruction (44), neuronal growth factor (NGF) (45), brain-derived neurotrophic factor (BDNF) (46), F wave and H reflex assessments related to spinal anterior horn motor neuron excitability during cross-inhibition, pro-vascular endothelial growth factor (VEGF) (47), inflammatory factors including TNF, IL-6, and NF-κB signaling pathways. Additionally, it is important to examine biological characteristics such as spastic muscle fiber thickness, muscle bundle length, pinnate angle, and the proportion of type I/II muscle fibers post-acupuncture (48, 49), By integrating insights from the central nervous system, peripheral nervous system, blood circulation system, skeletal muscular system, and neuro-immune-endocrine system, we can elucidate the theoretical connotation underlying acupuncture’s local-central-target organ-peripheral correlation effect. (3) Spastic hemiplegia can be categorized into various forms, including hand dysfunction, upper limb spastic hemiplegia, lower limb spastic hemiplegia, and foot drop. Currently, there is no systematic treatment protocol for selecting wave patterns and parameters of antagonistic muscles in different regions of the hands, feet, upper limbs, and lower limbs. In the future, the multi-center large-sample randomized controlled trials should be carried out to determine the guidance scheme for the selection of parameters such as wave mode and frequency of antagonistic muscle muscles in different parts of the hand, foot, upper and lower limbs.

## 6 Conclusion

The findings of this meta-analysis indicate that the integration of electroacupuncture treatment with antagonist muscle rehabilitation training can effectively alleviate muscle spasms, reduce muscle tension, and enhance lower limb motor function as well as daily living abilities. Although the improvement in various indicators was more pronounced in the treatment group compared to the control group, confounding factors, selection bias, and inherent limitations hinder a definitive interpretation of the safety and efficacy of electroacupuncture combined with rehabilitation training for spastic hemiplegia following stroke. This study offers preliminary evidence regarding the potential safety and efficacy of electroacupuncture in conjunction with antagonist muscle rehabilitation training for patients experiencing spastic hemiplegia post-stroke. Consequently, the findings of this study should be regarded as preliminary and exploratory; further validation through large-scale, long-term randomized controlled trials is essential.

## Data Availability

The original contributions presented in the study are included in the article/supplementary material, further inquiries can be directed to the corresponding author/s.
