# Supplementary figures and images for: Clinical efficacy of electroacupuncture antagonistic muscles combined with rehabilitation training in the treatment of spastic hemiplegia after stroke: a systematic review and meta-analysis of randomized controlled trials
Source: Front Neurol. 2025 Aug 8;16:1634845. doi: 10.3389/fneur.2025.1634845 (PMC12370752; doi:10.3389/fneur.2025.1634845)

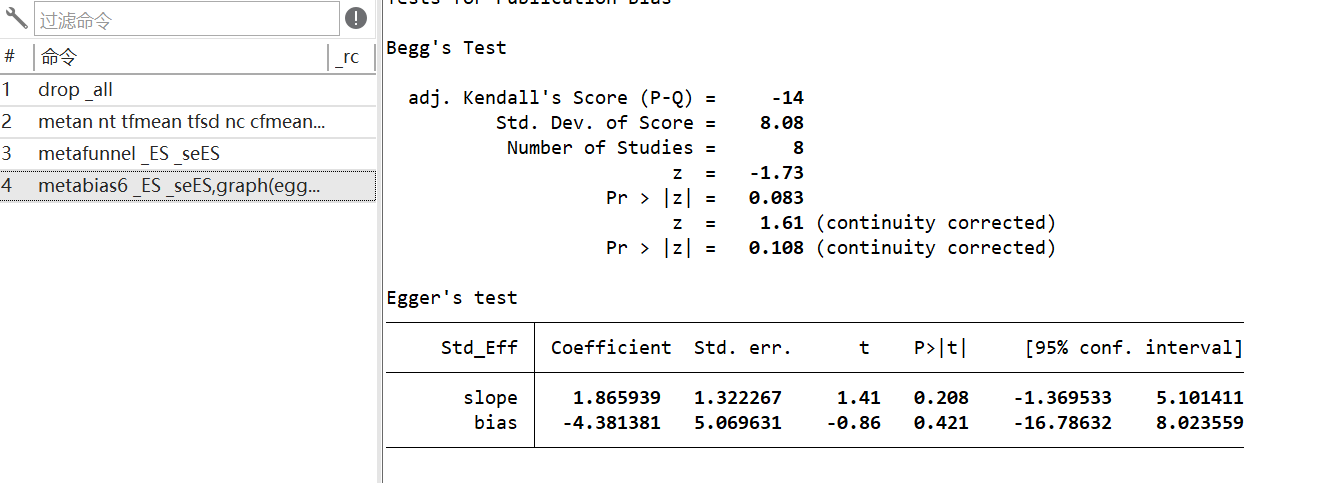

Supplement: Supplementary file 1 [file Data_Sheet_1.ZIP › Barthel Index funnel plot data.png]

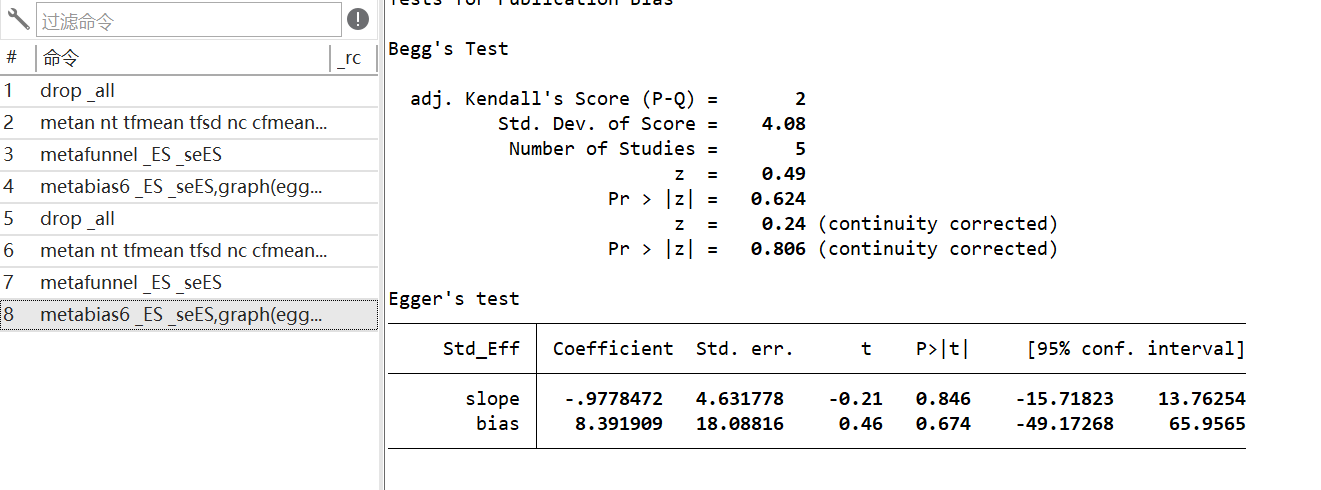

Supplement: Supplementary file 1 [file Data_Sheet_1.ZIP › Fugl-Meyer Assessment funnel plot data.png]

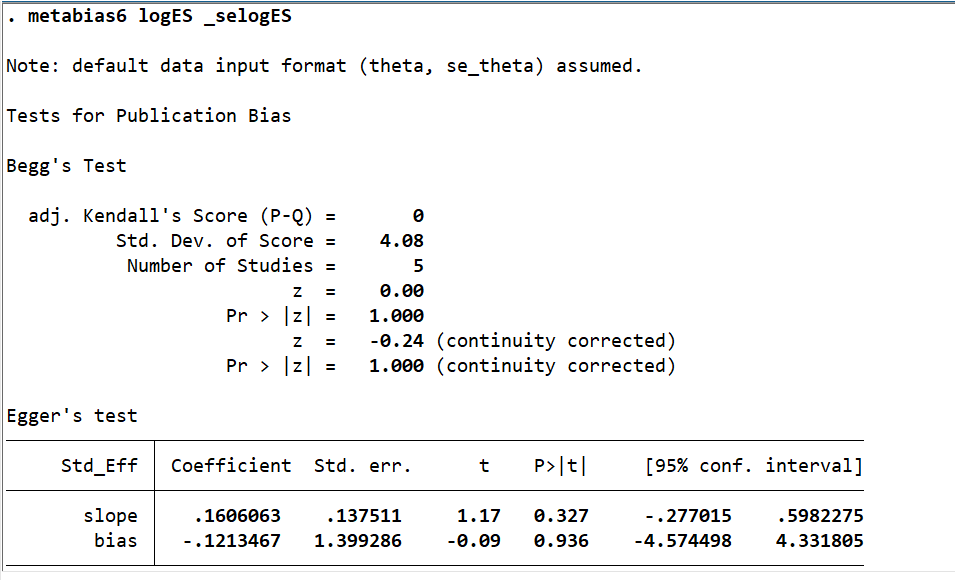

Supplement: Supplementary file 1 [file Data_Sheet_1.ZIP › Total efficiency funnel plot data.png]
