# Supplementary material for: Clinical efficacy of electroacupuncture antagonistic muscles combined with rehabilitation training in the treatment of spastic hemiplegia after stroke: a systematic review and meta-analysis of randomized controlled trials
Source: Front Neurol. 2025 Aug 8;16:1634845. doi: 10.3389/fneur.2025.1634845 (PMC12370752; doi:10.3389/fneur.2025.1634845)
Supplement: Supplementary file 2 [file Table_1.DOC]

| CBM search formula, as of March 27,2025 279 articles (("电针"[不加权:扩展]) OR ("电针干预"[常用字段:智能] OR "针刺加电针"[常用字段:智能] OR "电针刺激"[常用字段:智能] OR "电针结合"[常用字段:智能] OR "常规电针"[常用字段:智能] OR "电针治疗"[常用字段:智能])) AND (("随机对照实验"[常用字段:智能] OR "随机对照研究"[常用字段:智能] OR "RCT"[常用字段:智能] OR "随机"[常用字段:智能] OR "随机对照"[常用字段:智能]) OR ("随机对照试验"[不加权:扩展])) AND (("半身不遂"[常用字段:智能] OR "偏枯"[常用字段:智能] OR "中风偏瘫"[常用字段:智能] OR "偏风"[常用字段:智能] OR "脑卒中偏瘫"[常用字段:智能] OR "痉挛型偏瘫"[常用字段:智能] OR "肌张力增强"[常用字段:智能] OR "中风后偏瘫"[常用字段:智能] OR "肌张力增高"[常用字段:智能]) OR ("偏瘫"[不加权:扩展])) |
| --- |

CNKI search formula, as of March 27,2025 400 articles

（主题：痉挛性偏瘫）OR（主题：半身不遂）OR（主题：偏枯）OR（主题：中风偏瘫）OR（主题：偏风）OR（主题：脑卒中偏瘫）OR（主题：痉挛型偏瘫）OR（主题：肌张力增强）OR（主题：中风后偏瘫）OR（主题：肌张力增高）AND（主题：电针）OR（主题：电针干预）OR（主题：针刺加电针）OR（主题：电针刺激）OR（主题：电针结合）OR（主题：电针治疗）OR（主题：常规电针）AND（主题：电针）OR（主题：电针干预）OR（主题：针刺加电针）OR（主题：电针刺激）OR（主题：电针结合）OR（主题：电针治疗）OR（主题：常规电针）AND（摘要：随机对照试验(精确)）OR（摘要：随机对照实验(精确)）OR（摘要：随机对照研究(精确)）OR（摘要：RCT(精确)）OR（摘要：随机(精确)）OR（摘要：随机对照(精确)）

CSPD search formula, as of March 27,2025 758 articles

主题:(痉挛性偏瘫 or 半身不遂 or 偏枯 or 中风偏瘫 or 偏风 or 脑卒中偏瘫 or 痉挛型偏瘫 or 肌张力增强 or 中风后偏瘫 or 肌张力增高) and 主题:(电针 or 电针干预 or 针刺加电针 or 电针刺激 or 电针结合 or 常规电针 or 电针治疗) and 主题:(随机对照试验 or 随机对照实验 or 随机对照研究 or RCT or 随机 or 随机对照)

CCD search formula, as of March 29,2025 176 articles

[(((((((((((题名或关键词=痉挛性偏瘫 OR 题名或关键词=半身不遂) OR 题名或关键词=偏枯) OR 题名或关键词=中风偏瘫) OR 题名或关键词=偏风) OR 题名或关键词=脑卒中偏瘫) OR 题名或关键词=痉挛型偏瘫) OR 题名或关键词=肌张力增强) OR 题名或关键词=中风后偏瘫) OR 题名或关键词=肌张力增高) AND ((((((题名或关键词=电针 OR 题名或关键词=电针干预) OR 题名或关键词=针刺加电针) OR 题名或关键词=电针刺激) OR 题名或关键词=电针结合) OR 题名或关键词=常规电针) OR 题名或关键词=电针治疗)) AND (((((摘要=随机对照试验 OR 摘要=随机对照实验) OR 摘要=随机对照研究) OR 摘要=RCT) OR 摘要=随机) OR 摘要=随机对照))](https://qikan.cqvip.com/Qikan/search/index?LngMySearHistoryIdGuid=01a87094-b2c1-447d-aa17-1d097d02ce74&from=Qikan_Article_History)

Chinese Clinical Trial Registry Search as of March 29,2025 0 article

PUBMED search formula, as of March 30,2025 17 articles

((("Hemiplegia"[Mesh]) OR ((((((((((((((((((((((((((((Hemiplegias[Title/Abstract]) OR (Monoplegia[Title/Abstract])) OR (Monoplegias[Title/Abstract])) OR (Hemiplegia, Crossed[Title/Abstract])) OR (Crossed Hemiplegia[Title/Abstract])) OR (Crossed Hemiplegias[Title/Abstract])) OR (Hemiplegias, Crossed[Title/Abstract])) OR (Hemiplegia, Flaccid[Title/Abstract])) OR (Flaccid Hemiplegia[Title/Abstract])) OR (Flaccid Hemiplegias[Title/Abstract])) OR (Hemiplegias, Flaccid[Title/Abstract])) OR (Hemiplegia, Infantile[Title/Abstract])) OR (Hemiplegias, Infantile[Title/Abstract])) OR (Infantile Hemiplegia[Title/Abstract])) OR (Infantile Hemiplegias[Title/Abstract])) OR (Hemiplegia, Post-Ictal[Title/Abstract])) OR (Hemiplegia, Post Ictal[Title/Abstract])) OR (Hemiplegias, Post-Ictal[Title/Abstract])) OR (Post-Ictal Hemiplegia[Title/Abstract])) OR (Post-Ictal Hemiplegias[Title/Abstract])) OR (Hemiplegia, Spastic[Title/Abstract])) OR (Hemiplegias, Spastic[Title/Abstract])) OR (Spastic Hemiplegia[Title/Abstract])) OR (Spastic Hemiplegias[Title/Abstract])) OR (Hemiplegia, Transient[Title/Abstract])) OR (Hemiplegias, Transient[Title/Abstract])) OR (Transient Hemiplegia[Title/Abstract])) OR (Transient Hemiplegias[Title/Abstract]))) AND (("Electroacupuncture"[Mesh]) OR (Electroacupuncture[Title/Abstract]))) AND (randomized controlled trial[Publication Type] OR randomized[Title/Abstract] OR placebo[Title/Abstract])

Web of science search formula, as of March 30,2025 7 articles

TS=(Hemiplegia OR hemiplegics OR Monoplegia OR monoplegia OR Hemiplegia, crosseduncrossed Hemiplegia OR Crossed hemiplegics OR hemiplegics, Crossed OR Hemiplegia, Flaccid OR Flaccid Hemiplegia OR Flaccid hemiplegics OR hemiplegics, Flaccid OR Hemiplegia, Infantile OR hemiplegics, Infantile OR Infantile Hemiplegia OR Infantile hemiplegics OR Hemiplegia, Post-Ictal OR Hemiplegia, Post Ictal OR hemiplegics, Post-Ictal OR Post-Ictal Hemiplegia OR Post-Ictal hemiplegics OR Hemiplegia, Spastic OR hemiplegics, Spastic OR Spastic Hemiplegia OR Spastic hemiplegics OR Hemiplegia, Transient OR hemiplegics, Transient OR Transient Hemiplegia OR Transient hemiplegics)

TS=(Hemiplegia OR Hemiplegias OR Monoplegia OR Monoplegias OR Hemiplegia, CrossedORCrossed Hemiplegia OR Crossed Hemiplegias OR Hemiplegias, Crossed OR Hemiplegia, Flaccid OR Flaccid Hemiplegia OR Flaccid Hemiplegias OR Hemiplegias, Flaccid OR Hemiplegia, Infantile OR Hemiplegias, Infantile OR Infantile Hemiplegia OR Infantile Hemiplegias OR Hemiplegia, Post-Ictal OR Hemiplegia, Post Ictal OR Hemiplegias, Post-Ictal OR Post-Ictal Hemiplegia OR Post-Ictal Hemiplegias OR Hemiplegia, Spastic OR Hemiplegias, Spastic OR Spastic Hemiplegia OR Spastic Hemiplegias OR Hemiplegia, Transient OR Hemiplegias, Transient OR Transient Hemiplegia OR Transient Hemiplegias)

The Cochrane library search formula was screened to 31 literatures by April 3,2025, 10 evaluations were excluded, and 21 randomized controlled trials were included.

Search Name:

Date Run: 03/04/2025 19:11:54

Comment:

ID Search Hits

#1 hemiplegia 3441

#2 （Monoplegia）：ab,ti,kw OR （Monoplegias）：ab,ti,kw OR （Hemiplegia, Crossed）：ab,ti,kw OR （Crossed Hemiplegia）：ab,ti,kw OR （Crossed Hemiplegias）：ab,ti,kw OR （Hemiplegias, Crossed）：ab,ti,kw OR （Hemiplegia, Flaccid）：ab,ti,kw OR （Flaccid Hemiplegia）：ab,ti,kw OR （Flaccid Hemiplegias）：ab,ti,kw OR （Hemiplegias, Flaccid）：ab,ti,kw OR （Hemiplegia, Infantile）：ab,ti,kw OR （Hemiplegias, Infantile）：ab,ti,kw OR （Infantile Hemiplegia）：ab,ti,kw OR （Infantile Hemiplegias）：ab,ti,kw OR （Hemiplegia, Post-Ictal）：ab,ti,kw OR （Hemiplegia, Post Ictal）：ab,ti,kw OR （Hemiplegias, Post-Ictal）：ab,ti,kw OR （Post-Ictal Hemiplegia）：ab,ti,kw OR （Post-Ictal Hemiplegias）：ab,ti,kw OR （Hemiplegia, Spastic）：ab,ti,kw OR （Hemiplegias, Spastic）：ab,ti,kw OR （Spastic Hemiplegia）：ab,ti,kw OR （Spastic Hemiplegias）：ab,ti,kw OR （Hemiplegia, Transient）：ab,ti,kw OR （Hemiplegias, Transient）：ab,ti,kw OR （Transient Hemiplegia）：ab,ti,kw OR （Transient Hemiplegias）：ab,ti,kw 55

#3 #1 OR #2 3496

#4 Electroacupuncture 4005

#5 （Electroacupuncture）：ab,ti,kw 0

#6 #4 OR #5 4005

#7 (randomized controlled trial):ab,ti,kw OR (randomized):ab,ti,kw OR (placebo):ab,ti,kw OR (RCT):ab,ti,kw 1337509

#8 #3 AND #6 AND #7 31

Embase Retrieved as of April 4, 2025 22 articles

#6 #3 AND #4 AND #5 22

#5 'randomized controlled trial':ab,ti OR 'randomized':ab,ti OR 'placebo':ab,ti OR 'rct':ab,ti 1,283,050

#4 'electroacupuncture' 12,162

#3 #1 OR #2 29,045

#2 'hemiplegia, crossed':ab,ti OR 'crossed hemiplegia':ab,ti OR 'crossed hemiplegias':ab,ti OR 'hemiplegias, crossed':ab,ti OR 'hemiplegia, flaccid':ab,ti OR 'flaccid hemiplegia':ab,ti OR 'flaccid hemiplegias':ab,ti OR 'hemiplegias, flaccid':ab,ti OR 'hemiplegia, infantile':ab,ti OR 'hemiplegias, infantile':ab,ti OR 'infantile hemiplegia':ab,ti OR 'infantile hemiplegias':ab,ti OR 'hemiplegia, post-ictal':ab,ti OR 'hemiplegia, post ictal':ab,ti OR 'hemiplegias, post-ictal':ab,ti OR 'post-ictal hemiplegia':ab,ti OR 'post-ictal hemiplegias':ab,ti OR 'hemiplegia, spastic':ab,ti OR 'hemiplegias, spastic':ab,ti OR 'spastic hemiplegia':ab,ti OR 'spastic hemiplegias':ab,ti OR 'hemiplegia, transient':ab,ti OR 'hemiplegias, transient':ab,ti OR 'transient hemiplegia':ab,ti OR 'transient hemiplegias':ab,ti 997

#1 'hemiplegia'/exp OR hemiplegia 29045

English Clinical Trial Registry

No records have been found as of April 4, 2025
